# ATLAS—a psychobiological model of teacher stress in classroom interactions

**DOI:** 10.3389/fpsyg.2026.1869744

**Published:** 2026-06-19

**Authors:** Alexander Wettstein, Ida Schneider, Gabriel Jenni, Sonia J. Lupien

**Affiliations:** 1 Department of Research and Development, University of Teacher Education Bern, Bern, Switzerland; 2 Centre for Studies on Human Stress, Department of Psychiatry, University of Montreal, Montreal, QC, Canada

**Keywords:** allostatic load, classroom interactions, psychological strain, student development, teacher stress

## Abstract

Teachers are a particularly stressed occupational group with above-average burnout rates compared to other professions. Teacher stress significantly challenges teachers’ health, classroom interactions, and positive student development. Preventing this stress is of utmost importance. Proven frameworks help us understand and buffer teacher stress. However, each approach has its specific strengths and limitations in understanding the complex problem of teacher stress. We propose the comprehensive ATLAS Model of Psychobiological Teacher Stress in Classroom Interactions that builds on and extends existing frameworks. ATLAS considers objective measures, biological and interactional processes, the temporal dimensions of acute and chronic stress, and the effects of cumulative stress. The ATLAS Model permits us to understand (1) how teacher stress impairs teachers’ ability to provide an effective learning environment and (2) how this affects students’ learning and social development. (3) Finally, the model describes how teacher and student behavior shape dysfunctional classroom environments. A better understanding of the interplay between psychobiological stress in teachers, the classroom environment, and student development may help teacher education, schools, and healthcare providers mitigate adverse health outcomes and ultimately improve education quality.

## 1 Introduction

Compared to other professions, teachers report higher than average levels of self-perceived stress in the workplace (Kush et al., 2022; Redín and Erro-Garcés, 2020; Unterbrink et al., 2007) and burnout (Schaufeli, 2003). 46% of US teachers (Gallup, 2014) report high daily stress during the school year. Teacher stress negatively affects teachers’ mental and physical health (Skaalvik and Skaalvik, 2017). Work stress is associated with burnout (Maslach et al., 2001), depression (Ahola and Hakanen, 2007), and somatic complaints (Madigan et al., 2023). Teacher stress negatively impacts their ability to provide an effective learning environment. Teachers with high levels of psychological strain seem to struggle more than their colleagues to establish supportive teacher-student relationships and to manage classrooms effectively (Chang, 2009). Further, teacher stress affects students’ learning and social development. School is a central developmental context for promoting positive youth development and wellbeing (Kiuru et al., 2020). Strained teacher-student relationships and low instructional quality hinder students’ wellbeing, healthy development, and learning (Darling-Hammond et al., 2020). Finally, teacher stress presents a risk to the quality of the educational system. Many teachers prematurely leave their profession or retire early due to stress (Kim et al., 2020). Many countries face a serious teacher shortage that jeopardizes the quality of the education system (UNESCO and International Task Force on Teachers for Education 2030, 2024). In conclusion, teacher stress is a complex phenomenon with far-reaching consequences on teachers’ health, classroom interactions, positive student development, and the entire educational system. However, this complexity is often underrepresented in research on teacher stress.

Historically, psychological stress models and biophysiological research paradigms have largely operated in isolation, creating a disciplinary divide where internal biological alterations and live social interactions are rarely evaluated in tandem. The ATLAS model addresses this conceptual isolation by unifying biological, psychological, and social processes into a singular, integrative framework designed for both empirical research and pedagogical practice.

This article aims to address that challenge. First, we will outline the strengths and limitations of existing concepts in stress research. Next, we will introduce the comprehensive *Psychobiological Model of Teacher Stress in Classroom Interactions* that meets the specific needs of the profession, taking into account the perspectives of teachers, students, and the resulting interactive system. Finally, we will discuss the future implications for both research and practice.

### 1.1 General stress frameworks and teacher-specific applications

*Mia has been teaching middle school for 20 years. She feels increasingly drained by the high interactional demands and is developing physical complaints. She has become more thin-skinned, finds students more exhausting than before, and feels that her authority is being challenged. However, she tries to hide her feelings. Mia asks David to do his math task. But he refuses to do the work. Mia’s mouth goes dry, her pulse quickens, and her muscles tense up. She reprimands David so harshly that the whole class is startled. David flinches. He wants to give up and run away. No matter how hard he tries, he cannot get the math right. He throws his book on the floor and yells,* “Kiss my ass.” *Mia sends David out of the classroom. The mood in the class sinks to rock bottom. Mia’s thoughts go round and round in her head, but she cannot muster the energy to tackle the problems actively. Relationships and classroom management are increasingly difficult, and she feels abandoned by the school administration and her colleagues.*

Mia suffers from long-term cumulative stress and is entangled within a self-reinforcing burnout cascade (Jennings and Greenberg, 2009) in which teacher stress can increase harsh and reactive behavioral management strategies, escalating student disruptive behaviors, resulting in further increases in teacher stress and creating a cycle of stress, ineffective classroom management, and challenging student behavior (Herman et al., 2020a). Mia’s situation can be explained with various general stress frameworks and teacher-specific applications (see Table 1). Each framework considers specific aspects of Mia’s situation. However, clinical phenomena are often so complex that a single discipline or framework is not sufficient to solve the problem.

**Table 1:** Stress frameworks, their mechanisms, measures, effects, and implications.

Framework	Mechanism	Measures	Effects	Implications
Job Demands-Resources Model (Demerouti et al., 2001)	Job resources mitigate the negative health impact of job demands	Self-reports Observer ratings of working conditions	Individual and organizational outcomes	Balance job demands with adequate job resources
Transactional Stress Theory (Lazarus and Folkman, 1984)	Primary, secondary appraisal, and coping	Self-reports	Level of stress experienced is subjective	Modify maladaptive appraisals and teach new coping skills
Emotion and Emotional Labor (Kariou et al., 2021)	Surface acting versus deep acting	Self-reports Ecological Momentary Assessment	Surface acting leads to emotional exhaustion	Foster emotional regulation that promotes deep acting
The Prosocial Classroom Model (Jennings and Greenberg, 2009)	Teacher’s social and emotional competence and well-being are fundamental for positive classroom and student outcomes	Self-reports Observations	Improved teacher well-being, classroom climate, and student development	Teacher well-being is essential for creating healthier, more effective learning environments
Coping-Competence-Context (3C) Theory of Teacher Stress (Herman et al., 2020b)	Three pathways: coping, competence, and context	Self-reports Observations	Poor coping skills or unsupportive contexts leads to emotional exhaustion and bad instruction quality	Foster coping, classroom management, and systemic support
Model of Teacher Stress (Kyriacou and Sutcliffe, 1978)	Stress as a transactional, cyclical process that results in a response syndrome of negative affect	Self-reports Observations Physiological measures	Negative emotional affects and physiological and biochemical changes	Focus on appraisal, coping, and support systems
Transdisciplinary Model of Stress (Epel et al., 2018)	Stress as an interaction of individual, social, and biological factors	Self-reports Biomarkers to assess the allostatic load index	Cumulative stress influences how an individual responds to new stressors	Prevent cumulative stress that drives to negative health outcomes
ATLAS – A Psychobiological Model of Teacher Stress in Classroom Interactions	Reciprocal interaction between psychobiological teacher stress, classroom interaction, and student development	Self-reports Observations Physiological measures Biomarkers to assess the allostatic load index	Cumulative stress jeopardizes teacher perception, classroom interaction and student development	Prevent cumulative stress, foster teachers coping skills, classroom management and support within the school team

*The job demands-resources model* (Demerouti et al., 2001) examines how job demands and job resources influence occupational health. Teaching is highly demanding, ranging from addressing individual students’ needs and preventing classroom disruptions to handling student aggression, performing emotional labor in front of a class, managing classroom behavior, creating positive classroom ecologies, dealing with work pressure, meeting educational standards, and working with parents and colleagues. However, the individual’s psychological evaluation processes remain in a black box. It is unclear how teachers perceive work-related characteristics in the context of their idiosyncratic psychological processes. Thus, the strict dualism between demands and resources obscures how individuals deal with both.

*The transactional stress theory* of Lazarus and Folkman (1984) specifically focuses on how individuals subjectively appraise the significance of a situation based on their coping resources. Thus, instead of defining stress objectively in terms of external stimuli, the theory emphasizes the cognitive process by which a person assesses a situation as potentially stressful, which can lead to a state of stress. This approach has important implications for stress management: rather than simply combating external stressors, changing individual assessments and strengthening coping resources can be an effective strategy for managing stress (Li and Ma, 2025). While this model remains a cornerstone of psychological stress research, it is subject to several critical limitations. Primarily, it largely neglects the biological pathways of the stress response. Furthermore, reliance on self-reported data introduces memory and social desirability bias. Finally, as a general framework, the theory fails to account for the specific demands of the teaching profession and the unique environmental complexities of the classroom.

*The teacher emotion and emotional labor* literature considers teaching a highly demanding, public task and investigates the consequences of surface and deep acting on job satisfaction and burnout (Kariou et al., 2021). Surface acting, which involves suppressing authentic feelings to meet professional display rules, is consistently identified as a significant predictor of emotional exhaustion and depersonalization (Grandey and Melloy, 2017). Conversely, the genuine expression of positive emotions is adaptive for teacher wellbeing (Wang et al., 2019). Although this framework offers critical insights into the “cost of caring,” it is constrained by several limitations. By centering on how teachers regulate their internal states, the model risks pathologizing burnout as a deficit in individual emotional management, thereby obscuring the systemic stressors, such as excessive workloads, inadequate administrative support, and precarious school climates, that necessitate intensive emotional labor (Kariou et al., 2021). Furthermore, it relies on self-reports and neglects interactional processes and possible reciprocal emotional contagion between teachers and students.

*The prosocial classroom model* (Jennings and Greenberg, 2009) is not a framework of stress but rather a framework of teachers’ social–emotional competence and classroom climate. It accounts for the critical role of a teacher’s social and emotional competence and wellbeing in creating a positive, supportive learning environment for students. The model posits that a teacher’s ability to manage their own emotions and relationships, coupled with effective classroom management and social and emotional learning practices, leads to a healthier classroom climate that fosters students’ social, emotional, and academic growth.

*The coping-competence-context (3C) theory of teacher stress* (Herman et al., 2020b) is grounded in the Prosocial Classroom Model and focus on three critical interconnected pathways: Coping (e.g., active problem solving), Competence (e.g., classroom management), and Context (e.g., support within the school team) and links teacher stress to adverse outcomes for both teachers and students, providing a comprehensive framework for research and practice. The 3C Theory bridges individual psychology and organizational sociology, moving beyond individual-centered deficit models by recognizing that even highly competent teachers can struggle in a toxic work environment. However, like any theoretical framework, it faces methodological limitations. Most empirical studies using the 3C model rely on self-reports, which introduces subjectivity bias. Teachers may overestimate their Competence due to social desirability or underestimate their Coping resources during burnout. The high interconnectivity between Coping, Competence, and Context also makes it difficult to identify the most effective entry point for intervention. Finally, the model struggles to distinguish between a genuine skill deficit (“cannot do”) and stress-induced performance inhibition (“too stressed to do”), a distinction that is critical for determining whether a teacher needs professional training or psychological support.

*The model of teacher stress* (Kyriacou and Sutcliffe, 1978) conceptualizes stress as a sequence of events from potential stressors in the environment (e.g., student misbehavior), to the teacher’s appraisal of threat, the subsequent emotional and physiological response, and the applied coping strategy leading to a positive or negative outcome. Like transactional stress theory, this framework emphasizes the subjective assessment of a stressor and focuses primarily on the psychological and emotional aspects of the stress process. Moreover, physiological reactions such as increased heart rate and cortisol release are already accounted for in the model as side effects or consequences of the emotional stress reaction.

*The transdisciplinary model of stress* (Epel et al., 2018) unifies biological and psychological perspectives by describing stress as a set of interactive, emergent processes. A central feature of this model is *allostatic overload* (Juster et al., 2010; McEwen and Stellar, 1993), which refers to the stress-induced disruption of multiple biological systems to a permanently high, low, or non-adaptive state, even when the stressors subside. It impairs the ability to adapt to and respond effectively to the environment. The framework accounts for the cumulative nature of stress exposure and its impact on long-term health, conceptualizing chronic stress (psychological strain and allostatic load) as a crucial contextual factor that fundamentally alters the acute stress response.

Each of these frameworks has specific strengths and limitations in understanding the complex problem of teacher stress. The ATLAS framework builds on and extends these models, trying to holistically display the complexity of psychobiological teacher stress embedded in social interactions. ATLAS pays particular attention to the following four aspects:

1. *Subjective vs objective:* most frameworks assess an individual’s subjective appraisal with self-reports, while neglecting the objective work situation. ATLAS combines self-reports, observations, and biological markers to assess how teachers’ stress shapes classroom interactions, and how these, in turn, shape teachers’ and students’ wellbeing and health. We hypothesize that objective classroom environment measures offer a critical, complementary perspective that may account for variance in biological teacher stress and student development not fully captured by subjective self-reports.
2. *Biological processes:* teacher stress research primarily relies on self-reports and neglects biological processes (Herman et al., 2020b). From an evolutionary perspective, stress is an organism’s biological adaptive response to a threat or challenge from the environment. ATLAS examines teachers’ biological stress responses and their association with psychological processes and resulting behavior. However, because most biological processes are unconscious, we expect only weak associations between teachers’ biological and self-reported psychological processes.
3. *Interactions:* ATLAS examines the reciprocal relationships between psychobiological teacher stress, classroom interaction, and students’ learning and social development (Herman et al., 2020b). We expect that (1) teachers’ chronic stress, previous experience, personality, and mental filters shape their perception of the classroom environment, their regulation attempts, their behavior, and ultimately impair their ability to provide effective learning and social environments, and (2) subsequently jeopardize students’ learning and social development. 3) We further expect that the observed characteristics of the classroom environment profoundly impact both teachers’ and students’ wellbeing and health.
4. *Temporal dimensions of acute and chronic stress:* many models neglect the temporal distinction between acute and chronic stress and almost exclusively focus on acute stress reactions. Based on the Transdisciplinary Model of Stress (Epel et al., 2018), we conceptualize cumulative chronic stress as a crucial contextual factor that fundamentally alters the acute stress response. ATLAS considers the influence of psychological strain and allostatic load on teachers’ perceptions, regulation attempts, behavior, and evolving classroom interactions and student development.

In sum, the *ATLAS Model of Psychobiological Teacher Stress in Classroom Interactions* (briefly ATLAS Model) accounts for biological, psychological, and observational measures, uncovers the complex association between psychological and biological variables, examines the reciprocal relationships between psychobiological teacher stress, classroom interaction, and students’ learning and social development, and considers the temporal layering of acute and chronic processes, and the influence of cumulative stress and teachers’ prior experience on the acute stress response.

## 2 ATLAS—a psychobiological model of teacher stress in classroom interactions

ATLAS derives from “Associations between Teachers’ Psychological Strain and Allostatic Load, the Classroom Environment, and Student Development ATLAS”. Healthy teachers are an indispensable prerequisite for successful education. Like Atlas, the hero of Greek mythology who holds the celestial spheres, healthy teachers are the pillar of education.

**Figure 1:**
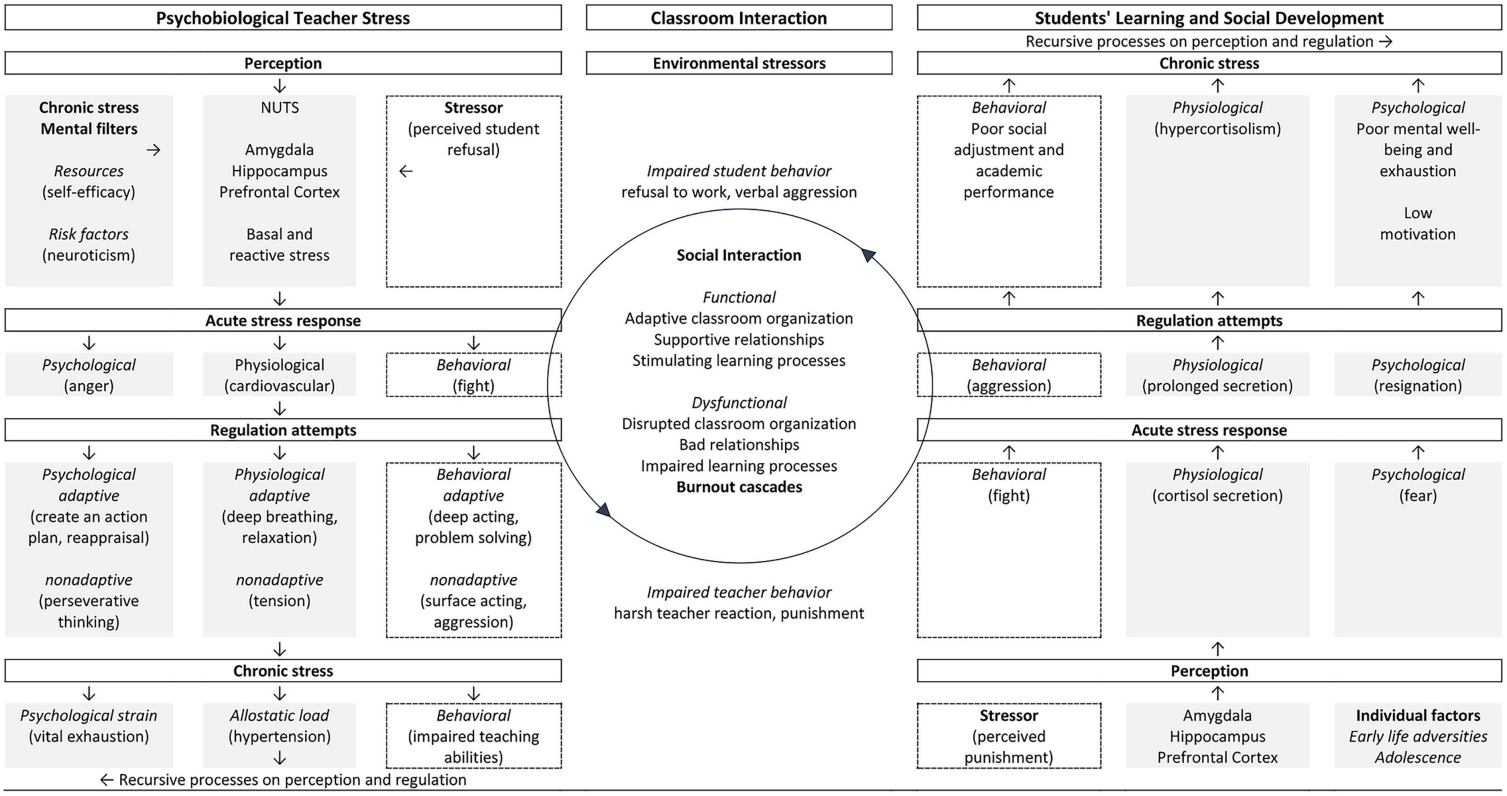
ATLAS – a psychobiological model of teacher stress in classroom interactions. Based on Wettstein (2012). This schematic representation provides a foundational overview, offering illustrative examples in parentheses to enhance understanding.

The ATLAS model (see Figure 1) consists of three columns, each introduced in a separate section. Section 2.1 describes teachers’ acute psychobiological stress response, the influence of previous experience and habitual mental filters, teachers’ regulation attempts, and the resulting psychological strain and allostatic load (left column). Section 2.2 shows how teacher stress alters classroom interaction (middle column), while Section 2.3 examines how altered classroom systems affect students (right column). Finally, Section 2.4 demonstrates how systemic cascades and burnout loops are characterized by dysfunctional interaction processes that affect both teachers and students (center of the model).

### 2.1 Teacher’ psychobiological stress

We start with the teacher perspective and show how teachers’ chronic stress, previous experience, and mental filters shape their perception of the classroom environment, their regulation attempts, and their behavior, and impair their ability to provide effective learning environments. We delve into physiological processes before moving on to psychological factors. This order is mainly based on evolutionary theory, which suggests that biological systems emerged first, followed by psychological and social-interactional systems.

From an evolutionary perspective, acute stress is an organism’s adaptive response to environmental threats or challenges. These biological stress responses are accompanied by psychological processes and resulting behavior. The brain is the central organ of the stress response, and it specifies what is threatening and, thus, potentially stressful. The brain further elicits physiological and behavioral responses that can be either adaptive or damaging (McEwen, 2007, 2008). In the context of teaching, such environmental stressors can be wide-ranging (e.g., student misbehavior, parent-teacher conferences) and can be perceived as a threat by teachers.

Stressors can be *absolute* (a threat to survival, e.g., a serious physical attack by a stronger student that seriously endangers the physical integrity of the teacher, leading to a significant stress response in every person facing this threat) or they can be *relative* (a threat to the self; e.g., a student’s refusal to work, dependent on individual interpretation as student’s excessive demands at school or oppositional behavior that questions the teacher’s authority). In the case of relative stressors, four psychological components evoke a physiological stress response in humans: novelty, unpredictability, threat to the ego, and sense of low control (NUTS; Dickerson and Kemeny, 2004). Relative threats are interpreted according to the cognitive appraisal of situation demands and available resources. As such, stress is a highly personal experience because what is deemed stressful for one individual may not be for another.

An equally vital distinction lies between *basal* and *reactive* stress states. To navigate real-world challenges, organisms rely on an intrinsic physiological architecture that regulates essential daily homeostatic and basal somatic functions while remaining highly responsive to perceived environmental challenges. When a relative or absolute threat is perceived, the organism shifts from homeostatic equilibrium into a reactive stress state. Crucially, acute stress is not the stressor itself, but rather the organism’s highly adaptive biological mobilization designed to facilitate active coping. This adaptive response mobilizes the metabolic energy required to overcome immediate environmental challenges.

#### 2.1.1 The acute stress response

When an educator’s brain detects a relative or absolute threat, an acute stress response is elicited. This immediate reaction encompasses highly integrated physiological (e.g., hormonal, neurochemical), psychological (e.g., fear, anger), and behavioral (e.g., fight, flight, affiliation) dimensions (Laferton et al., 2023; Mauss and Robinson, 2009). To systematically delineate the architecture of the ATLAS model illustrated in Figure 1, these three components are introduced sequentially for analytical clarity, although in reality, they operate in parallel and continuously exert reciprocal influences on one another.

*The physiological stress response* involves a coordinated network of autonomic, neuroendocrine, metabolic, and immune subsystems. We concentrate on two interrelated systems here: (1) The *sympathetic adrenomedullary (SAM) axis* initiates the fight-or-flight response through the secretion of the catecholamine hormones (adrenaline and noradrenaline) within seconds of threat exposure from the adrenal medulla, as commanded by the hypothalamus’ perception of arousal. (2) The *hypothalamic–pituitary–adrenal (HPA) axis* is also activated by the hypothalamus within minutes after threat exposure. This physiological stress response mobilizes metabolic energy. Cortisol helps to mobilize energy through glucose metabolism at the expense of other systems (such as reproduction, immunity, inflammation, and growth), which are not immediately required to face acute stressors (Lupien et al., 2009). These physiological stress responses of the SAM and HPA axis have beneficial effects, particularly in the short term to cope with the stressor (Koolhaas et al., 2011; Sapolsky et al., 2000). The stress response mobilizes energy to fight danger, provides oxygen and nutrients to active organs and tissues, and changes behavior adaptively. The release of cortisol further increases memory of the stressful situation, which prepares individuals for future stressor encounters (Taborsky et al., 2022).

*The psychological stress response* varies depending on the level of perceived stress. Moderate stress increases performance and is often seen as a challenge. Severe stress reduces performance and can lead to a block in action. The National Scientific Council on the Developing Child (Harvard University, 2020) proposed the following simple taxonomy to describe three categories of stress experience: positive, tolerable, and toxic. Positive stress refers to moderate, short-term increases in blood pressure, heart rate, and stress hormones. Tolerable stress describes a physiological state that could potentially disrupt neuronal functioning but can be buffered by supportive relationships that facilitate adaptive coping. Toxic stress includes strong, frequent, or prolonged activation of the body’s stress management system. Stressful events that are chronic, uncontrollable, and/or experienced without having access to support tend to provoke these types of toxic stress responses (Harvard University, 2020).

*The behavioral stress response* refers to the following behavioral patterns: fight, flight, affiliation, appeasement, or fawning. Under stress, teachers may react aggressively (fight) or with social withdrawal (flight) to challenging student behavior. Affiliation, appeasement, and fawning are evolutionarily newer strategies. *Affiliation* with others is a basic human coping response when facing a threat (Taylor, 2011). Affiliating with others is beneficial for mental and physical health. *Appeasement* is a pacification and submission response that may serve to de-escalate a situation. The ability to access the appeasement process is conceptualized as a type of “super social engagement” that requires the neural capacity to manage a hybrid state that enables access to the calming and social cuing of the social engagement system (Porges, 2022), while simultaneously maintaining access to the energetic mobilization sympathetic system to engage fight/flight behaviors if necessary (Bailey et al., 2023; Porges, 2022). *Fawning* refers to people-pleasing with the aim of diffusing conflicts and earning the approval of others (Bailey et al., 2023).

Teachers’ physiological, psychological, and behavioral systems do not act independently; they interact continuously. Physiological stress reactions are accompanied by psychological responses. Glucocorticoids and catecholamines can lead to changes in cognitive processing (Lupien et al., 2015). Finally, a teacher’s physiological and psychological processes influence their behavioral responses in social interactions, causing us to react more (support) or less adaptively (aggression or withdrawal) in challenging situations.

Within the overarching ATLAS framework, this tripartite acute response represents the dynamic nucleus of the model. When a classroom stressor activates the SAM and HPA axes, the resulting physiological shifts narrow cognitive bandwidth (psychological pathway) and promote ingrained behavioral tendencies (behavioral pathway). In live classroom interactions, this biological cascade directly precipitates a transition from proactive teaching to reactive strategies, such as harsh fight-responses or protective withdrawal, which subsequently degrades the socio-emotional climate and initiates student distress loops.

While the acute stress response shapes the immediate behavioral and physiological dynamics, these reactions are not uniform across individuals. The ATLAS model posits that these acute cascades are strictly filtered and pre-configured by a teacher’s cumulative past experiences and historical perceptual lenses, which are detailed in the following section.

#### 2.1.2 The influence of previous experience and habitual mental filters

We will now take a step back in the ATLAS Model to examine how previous experiences influence teachers’ perceptions. The nature of reality is subjective, and past experiences, available resources, and perceived risks all play a crucial role in shaping perception. Teachers perceive student behavior through the lens of their habitual mental filters, resources, and vulnerabilities. What we perceive is not an exact replica of the external world (Lupien et al., 2015). Modern views of the development of emotional and stress responses view the brain as a “prediction machine” where appraisals of events are shaped in part by one’s personal memory bank of what to expect, as well as by the current stimuli (Barrett and Simmons, 2015). Thus, teacher stress is not exclusively a reflection of external stressors but mirrors teachers’ personalities, prior stress experiences, and coping styles (Chan, 1998).

Studies show that teachers’ personality factors (e.g., neuroticism), maladaptive coping attempts (e.g., a high resignation tendency), and cumulative stress (e.g., chronic worry, emotional exhaustion) contribute to an increased perception of student misbehavior or aggression (Evers et al., 2004; Kokkinos et al., 2005; Tsouloupas et al., 2010; Wettstein et al., 2023a). This highlights that teachers’ perceptions of their environment are fundamentally influenced by individual resources and risk factors, such as personality, attitudes, and expectations (Kyriacou, 2001). Consequently, identifying potential individual risk and protective factors among these constructs is crucial to preventing teacher stress.

Neuroticism constitutes a critical risk factor within teacher stress trajectories (Kim et al., 2019; Kokkinos, 2007), a trait disposition to experience adverse emotions, including anger, anxiety, self-consciousness, irritability, emotional instability, and depression (Costa and McCrae, 1992). People with elevated neuroticism respond strongly to environmental stressors and interpret ordinary situations as threatening (Tomaka and Magoc, 2021). Teachers high in neuroticism have been found to perceive more classroom disruptions, which in turn lead to more occupational problems 2 years later (Jenni et al., 2025). In addition to neuroticism, there are a number of other personality factors, such as a hostile personality, anxiety trait, and hopelessness reactivity, that are associated with an increased release of stress hormones (for a review, see Kudielka et al., 2009).

On the other hand, core self-evaluation (CSE) is a personality factor that serves as a protective factor. CSE is a higher-order factor, including global self-esteem, generalized self-efficacy, emotional stability, and internal locus of control (Judge et al., 2002), protecting teachers against vital exhaustion (Schneider et al., 2022). Similarly, a meta-analysis suggests that CSE may be a protective factor in lowering an individual’s risk of burnout (Alarcon et al., 2009). A related construct is teacher self-efficacy, which encompasses how capable teachers perceive themselves in the classroom, especially in challenging situations. It is related negatively to teacher stress and burnout (Skaalvik and Skaalvik, 2017) and positively linked with students’ level of motivation (Zee and Koomen, 2016). Finally, the degree of satisfaction is a central protective factor and influences how favorably we perceive our environment. It is multifaceted and encompasses satisfaction with work, family, friends, oneself, and life in general. Individuals with high satisfaction view things more optimistically and actively create a more favorable environment supported by positive attitudes (Seligman, 2002). Accordingly, CSE is associated with higher job and life satisfaction (Chang et al., 2012; Judge et al., 2005). In contrast, individuals with high levels of neuroticism often exhibit lower levels of life satisfaction (DeNeve and Cooper, 1998). Finally, a high stress resistance, positive beliefs about stress, adaptive, flexible cognitions, and humor might cushion teachers’ stress (Lupien, 2020).

In addition to personality factors, attitudes and expectations can increase or alleviate stress. Negative stress beliefs (or “stress mindsets”) refer to specific attitudes and opinions individuals have regarding stress (Laferton et al., 2023). These may entail notions such as “stress makes me less productive” or “stress is bad for my health”. Thus, stress mindsets are the meta-cognitive processes that shape how individuals interpret stress’s overall meaning as debilitating (negative) or enhancing (positive). In teachers, negative stress mindsets predict job turnover (Kim et al., 2020).

#### 2.1.3 Regulation attempts

Teachers’ acute stress responses trigger psychological, physiological, and behavioral attempts at regulation in the medium term. *At a psychological level*, stress is not something that happens to us, but how we react to something. The choice of a specific emotion regulation strategy is a special case of decision-making. At this stage, stress refers to a transaction between a person and their environment in which demands are perceived as threatening while the resources for coping are considered insufficient (Friedman-Krauss et al., 2014; McCarthy et al., 2016). We distinguish between three broader coping styles (Wang et al., 2022). *Problem-focused coping* (e.g., analyze the problem and create an action plan) aims to modify the problem and is adaptive when individuals view a situation as controllable. In contrast, *emotion-focused coping* (e.g., acceptance) aims to reduce the intensity of unpleasant emotions and is adaptive when individuals appraise the situation as uncontrollable. Finally, *disengagement strategies* are maladaptive and characterized by perseverative thinking, self-blaming, denial, and resignation. Here, the cognitive unproductive confrontation with the stressor does not lead to a solution but rather intensifies the original problem. The case example of Mia described above also illustrates a maladaptive regulation attempt characterized by perseverative thinking without actively tackling the problems.

*At a physiological level,* adaptive strategies include deep breathing (which stimulates the vagal brake), exercise, and relaxation. Stress is nothing other than mobilized energy. It can, therefore, also be reduced through movement. In addition, increased arousal after sports is not attributed to the original stressor but to the sporting activity. In contrast, maladaptive regulation attempts include, like in Mia, strong muscular tension and continuously high arousal (Hoehn-Saric and McLeod, 2000). They severely impair thinking and the adaptive ability to act.

*At a behavioral level,* we differentiate between habitually adopting approach or avoidance strategies (Finset et al., 2002). Approach strategies, such as active problem-solving and seeking social support, are widely regarded as adaptive coping mechanisms. Research indicates that these strategies are particularly effective over the medium to long term and are inversely associated with emotional exhaustion (De Rijk et al., 1998).

Avoidance strategies, such as social withdrawal, are risk factors because the avoided problems remain unsolved. In the long run, this can reduce stress resilience since the unsolved problems continue to generate stress in the individual. Hence, avoidant coping strategies are likely to deplete resources and, in the long run, contribute to teachers’ stress experience and growing levels of emotional exhaustion (Ito and Brotheridge, 2003).

When teachers are confronted with challenging teaching situations, they can try to hide their true emotions or act authentically. *Surface acting* (hiding or faking emotions) requires constant and purposeful self-control and regulatory processes, depleting a substantial amount of teachers’ mental resources, and is positively related to higher hair cortisol levels (Qi et al., 2017), burnout, and lower job satisfaction (Yin et al., 2019). In contrast, with *deep acting,* teachers attempt to change their internal feelings to match the emotions they need to display. For example, they might cognitively reappraise a student’s refusal to work as being overwhelmed at school and subsequently act more authentically and meaningfully with students, which can improve the classroom environment and student motivation.

Under stress, some teachers are no longer able to make rational decisions and weigh up pedagogical alternatives. They react largely automatically, and there is a risk that they will fall into non-adaptive, stress-related patterns of action like withdrawal or teacher aggression that aggravate student misbehavior (Wettstein and Scherzinger, 2022). *Teacher aggression* damages social relationships and can lead to emotional problems such as depression and social withdrawal, aggression, and learning difficulties in students (Montuoro and Lewis, 2015; Wettstein, 2008). In contrast, some teachers are emotionally exhausted and withdraw, losing the capacity to lead the class appropriately, build a positive class climate, and meet the needs of individual students (Wettstein and Scherzinger, 2022). Teacher stress and maladaptive coping profiles predicted the use of reprimands later in the school year (Herman et al., 2020a).

*In sum*, looking at psychological and behavioral regulation attempts simultaneously, three coping profiles can be identified among teachers (Wang et al., 2022): *Adaptive copers* show high levels of problem-solving and seeking social support, with low use of disengagement strategies, resulting in positive outcomes. *Problem-avoidant copers* show low levels of problem-solving and support-seeking, combined with a high degree of problem avoidance, leading to poorer professional outcomes, low job satisfaction, high burnout levels, and intentions to quit the profession. *Social-withdrawal copers* exhibit high levels of disengagement and social withdrawal. This is consistently found to be a maladaptive profile associated with negative outcomes. Thus, teachers must be made aware of maladaptive attempts at regulation and supported in developing adaptive coping styles. Proper regulation of the stress system is crucial, given that chronic activation of physiological stress responses is involved in various disorders such as anxiety and depression (Carnegie et al., 2014; Raymond et al., 2019; Schiefelbein and Susman, 2006; Vedhara et al., 2003) and jeopardizes social interactions within the classroom. Finally, the teaching profession is characterized by its fuzzy boundaries. There is always more that can be done. That is why it is important for teachers to detach and recover in their free time.

#### 2.1.4 When stress becomes chronic: teachers’ psychological strain and allostatic load

In the long term, stress can result in a variety of psychological and physiological costs that have a profound impact on affected teachers, as illustrated in Mia’s case example. While chronic stress is often caused by work, it affects both work and life in general, e.g., regarding the demands of family life or personal challenges. Prolonged stress exposure without sufficient recovery falters physiological regulation and might lead to an allostatic overload (see below; Juster et al., 2010). In addition, such physiological consequences of stress might go unnoticed. Consequently, understanding work-related causes of stress and burnout, as well as various psychological and physiological consequences, is crucial to counteract teacher stress.

Prolonged exposure to stressors can lead to psychological strain. Psychological strain can manifest in different areas of life (e.g., at work, regarding the self, or in one’s private life; Hagemann and Geuenich, 2009). Occupational problems also include negative feelings related to work. Self-related problems include low frustration tolerance, feelings of depersonalization, or concentration problems. Family-related problems encompass estrangement or decreased participation in familial life. Friends-related problems include reduced interest in friends’ lives and retreating from social contact. Psychological strain can also include psychosomatic symptoms. Vital exhaustion is a psychosomatic state of unusual fatigue, lack of energy, irritability, and demoralization (Appels and Mulder, 1989). Vital exhaustion is a potential early warning sign of cardiovascular disease (Frestad and Prescott, 2017) and seems closely related to burnout (Schoch et al., 2018).

Teachers with high levels of psychological strain may struggle to manage classrooms effectively and to establish supportive teacher-student relationships (Herman et al., 2018). Teachers who reported higher levels of burnout were observed to use harsher reprimands and less praise, whereas teachers reporting higher levels of efficacy in classroom management used more proactive classroom management strategies (Reinke et al., 2013).

Multisystemic physiological dysregulations might result in an allostatic overload. Allostatic overload (Juster et al., 2010; McEwen and Stellar, 1993) refers to the perturbation of several physiological systems characterized by consistently high or low or non-adaptive states even when stressors remit. It affects teachers’ ability to adapt and effectively respond to the environment. Allostatic overload, i.e., the cumulative wear and tear on the body due to chronic stress, is measured using an allostatic load index, which reflects the activity of the cardiovascular (e.g., heart rate, heart rate variability, and blood pressure), neuroendocrine (e.g., hair cortisol), immunological (e.g., markers of inflammation), and metabolic (e.g., body mass index) systems (Juster et al., 2010). Essentially, the allostatic load index assesses how the body’s systems respond to repeated or prolonged stress and what effects these responses have on overall health.

In teachers, a reduction in blood pressure, static muscle tension, psychosomatic symptoms, and adrenaline levels occurs less during weekends and more during summer vacations (Ritvanen et al., 2003). Chronic work stress and exhaustion are associated with higher allostatic load in female school teachers (Bellingrath et al., 2009). Support from other teachers or school administration has a protective effect on teachers’ allostatic load (Wettstein et al., 2023b).

The brain’s short-term responses to novel and potentially threatening situations may be adaptive and result in new learning and acquired behavioral strategies for coping. Repeated stress, however, can cause both cognitive impairments and structural changes in the hippocampus. Cortisol can cross the blood–brain barrier and bind to glucocorticoid receptors, mostly concentrated in the prefrontal cortex, the amygdala, and the hippocampal formation (Lupien et al., 2015). The brain structures affected by cortisol also play a role in identifying and interpreting situations as being stressful (or threatening), and in the selection and/or inhibition of potential responses to these situations. Each of these processes may occur somewhat independently of the other and contribute in various degrees to different pathophysiological situations involving traumatic stress, depression, or aging (Lupien et al., 2015).

Chronic dysregulation in glucocorticoid levels has been associated with increased risk for depression and burnout, the two conditions showing, respectively, increased or decreased levels of cortisol (Lupien et al., 2015). Consequently, long-term stress in teachers can lead to allostatic overload and to the physiological and/or psychological disorders associated with it.

*In sum*, teacher stress results from the interplay among prior experience and mental filters, perceptions of the classroom environment, and attempts at regulation. For teachers like Mia, psychobiological stress impairs their ability to provide an effective learning environment.

Within the ATLAS framework, the acute psychobiological stress response and cumulative wear-and-tear represent the foundational processes of the model. When a classroom stressor activates the SAM and HPA axes, personality filters like CSE determine the initial threshold of threat perception. If stress becomes chronic, allostatic overload exerts a neurotoxic effect on prefrontal regions responsible for emotional control and behavioral flexibility. This biological erosion can alter teacher behavior by narrowing cognitive capacity, setting the stage for reactive, ingrained interaction tendencies in the classroom.

Having established the internal psychobiological state of the teacher, the ATLAS model posits that these hidden internal dynamics must manifest outwardly. The subsequent section delineates the precise interactional pathways through which a teacher’s internal stress profile alters live classroom management and reciprocal relational dynamics.

### 2.2 How stress alters classroom interaction

The internal psychobiological strain and the subsequent regulatory efforts of the educator do not remain hidden; instead, they directly alter the interpersonal dynamics of the classroom. To understand how these regulatory attempts visually map onto the interactional core of the ATLAS model (as illustrated in Figure 1), these behavioral manifestations must be systematically analyzed across three distinct, overlapping domains of the classroom ecology:

1. *The decline in proactive classroom management:* when a teacher’s internal psychobiological resources are compromised by acute or chronic stress, the cognitive bandwidth required to maintain complex, low-threshold instructional flow and proactive monitoring is restricted. Instead of utilizing adaptive, problem-focused coping styles to smoothly regulate minor disruptions, the teacher’s management practices shift toward reactive patterns. Under these conditions, teachers frequently display delayed or disproportionately harsh responses to student behavior, breaking instructional momentum, and increasing overall classroom restlessness.
2. *The dynamic of emotional labor and disengagement:* when teachers are confronted with challenging teaching situations, they can try to fake emotions or hide their true emotions (surface acting) or act authentically (deep acting). Surface acting requires constant and purposeful self-control, depleting a substantial amount of teachers’ mental resources, and is positively related to higher hair cortisol levels (Qi et al., 2017), emotional labor (Attwood, 2024), burnout, and lower job satisfaction (Yin et al., 2019). In contrast, with deep acting, teachers attempt to change their internal feelings to match the emotions they need to display, cognitively reappraising a student’s refusal to work as being overwhelmed, which improves student motivation. Under stress, some teachers are no longer able to make rational decisions. They react automatically, falling into non-adaptive actions like withdrawal or teacher aggression that aggravate student misbehavior (Wettstein and Scherzinger, 2022).
3. *Relational disruption and social ecologies:* proper regulation of the stress system and an adaptive coping style is crucial, given that chronic activation of physiological stress responses is involved in various disorders (Carnegie et al., 2014) and jeopardizes social interactions within the classroom. This deterioration directly fractures the balance of agency and communion between teacher and student, shifting the interaction from a shared social ecology to a fragmented space of relational conflict (Oldfield and Ainsworth, 2022).

From the ATLAS perspective, regulation attempts function as the manifest behavioral mediators that bridge a teacher’s internal psychobiological state with the shared classroom environment. While adaptive coping strategies preserve instructional quality, maladaptive regulation attempts, such as surface acting, teacher aggression, or social withdrawal, deplete psychological resources. This maladaptive behavioral shift directly degrades classroom organization and relational communion, actively transmuting individual psychological strain into a collective, dysfunctional classroom climate.

This degradation of instructional quality and relational safety does not remain contained within the teacher’s individual sphere. The ATLAS model specifies that this altered interaction system acts as a primary environmental stressor for the student body, significantly hindering student development and wellbeing, which constitutes the downstream consequences detailed in the following chapter.

### 2.3 How altered classroom systems affect students

We now shift our focus to the student population within the classroom ecology. Just like teachers, students also experience an acute stress response in threatening situations, which is influenced by their perception and individual factors. Mirroring the teacher’s processing architecture, students interpret their immediate environment through the background of their prior biographical experiences, available resources, and constitutional vulnerabilities. In recent years, empirical trends indicate a concerning decline in student mental health globally (Cosma et al., 2025), with youth wellbeing remaining intrinsically tied to proximal classroom climates and interpersonal dynamics (Collie and Hascher, 2024). Within the ATLAS framework, two foundational dimensions actively shape students’ individual psychobiological stress responses:

1. *Early life adversities*: the human brain serves as the primary organ of biological adaptation, continuously remodeling its structural and functional architecture in response to environmental experiences, including developmental exposures to chronic stress (McEwen, 2009). Children developing within harsh or unpredictable environments frequently acquire highly specialized, stress-adapted skills. While these adaptations enable youth to detect interpersonal threats more rapidly during social interactions (Ellis et al., 2022) and navigate volatile relational spaces, persistent exposure to threatening information inevitably conditions the cognitive system to respond hypersensitively. Over time, this hypervigilant architecture accelerates allostatic accumulation (McEwen, 1998), culminating in severe psychological (e.g., anxiety), biological (e.g., allostatic overload), and behavioral (e.g., disruptive or maladaptive) systemic tolls (Masten, 2001).
2. *Increased vulnerability in adolescence:* adolescence constitutes a critical developmental transition characterized by profound neuroendocrine, psychological, and social remodeling, alongside heightened performance pressures during the educational transition to secondary schooling (Raufelder et al., 2022). Navigating these multifaceted developmental demands, adolescents report escalating levels of school exhaustion linked to compounding academic expectations (Inchley et al., 2016; OECD, 2017; Pascoe et al., 2020). Empirically, elevated academic stress exhibits robust negative associations with academic achievement and powerful positive correlations with school dropout (Dupéré et al., 2018), internalizing mental health problems (Eppelmann et al., 2016; Snyder et al., 2019), and externalizing dissocial behaviors (Lüdeke and Linderkamp, 2021).

The empirical consequences of an altered classroom system and its influence on students’ health and stress outcomes can be systematically organized across three primary developmental domains:

1. *Impact on student learning and cognitive processing:* educator stress directly compromises instructional quality, thereby undermining student learning trajectories (Klusmann et al., 2016). From a psychobiological standpoint, teacher strain spills over into the student body; chronic exposure to an elevated stress-response environment affects students’ hippocampus, amygdala, and prefrontal cortex, inducing neurotoxic alterations that impair executive attention, memory consolidation, and emotional regulation (Lupien et al., 2009). Consequently, classrooms led by teachers reporting high stress and burnout, paired with low coping efficacy, exhibit significantly lower academic achievement scores (Herman et al., 2018).
2. *Impact on student motivation and school engagement:* aligning with the Prosocial Classroom Model (Jennings and Greenberg, 2009), an educator’s compromised socio-emotional capacity and depleted wellbeing directly degrade instructional execution and classroom management, generating adverse student outcomes. Meta-analytic evidence corroborates that teacher burnout is robustly associated with diminished student motivation, relational disengagement, and depressed academic performance (Madigan and Kim, 2021). When the instructional environment degrades due to teacher depletion, students manifest sharp declines in active task engagement and intrinsic motivation to perform.
3. *Socio-emotional vulnerabilities, distress, and behavior:* elevated teacher strain accelerates the deployment of harsh, reactive behavioral management strategies, which in turn escalates student disruptions, solidifying a dysfunctional relational cycle (Herman et al., 2020a). Moreover, educator stress impairs students’ broader social adjustment and emotional regulation capacities (Poulou and Denham, 2023). Classrooms managed by emotionally exhausted teachers exhibit markedly higher frequencies of student disruptive behaviors (Herman et al., 2018).

Longitudinal data demonstrate that students receiving predominantly negative interpersonal feedback from their teachers exhibit significant subsequent increases in emotional dysregulation, concentration deficits, and manifest behavioral disruptions by the end of the academic year, whereas a surplus of positive feedback predicts significant gains in prosocial behaviors (Reinke et al., 2016). Extensive research indicates that an educator’s emotional exhaustion, characterized by chronic emotional overstrain and the depletion of affective resources, directly compromises student wellbeing. Under these conditions, students perceive themselves as less cared for, report lower school satisfaction (Arens and Morin, 2016; Ramberg et al., 2020), exhibit elevated depressive symptomatology (Ishak et al., 2013), and manifest physiological distress, characterized by significantly elevated diurnal cortisol concentrations (Oberle and Schonert-Reichl, 2016).

Within the downstream arc of the ATLAS framework, the compromised behavior of an emotionally exhausted teacher acts as a direct environmental catalyst that alters student outcomes. Teacher psychological strain does not influence student development directly; rather, its impact is mediated by manifest, observable pedagogical behaviors. Chronic exposure to an emotionally depleted classroom climate precipitates “stress contagion,” elevating students’ own physiological stress markers (Oberle and Schonert-Reichl, 2016). This downstream pressure is heavily moderated by individual student vulnerabilities, causing hypervigilant or trauma-exposed youth to respond with elevated disruptive behaviors.

Crucially, the ATLAS framework does not view student outcomes as a terminal, unidirectional outcome. Having established how altered classroom environments manifest as compromised student learning, motivation, and socio-emotional strain, the final stage of our model completes the systemic loop, illustrating how these deteriorated student behaviors feed back into the teacher’s system, locking both parties into a self-reinforcing burnout cascade.

### 2.4 Systemic cascades and burnout loops

#### 2.4.1 Coercive escalations and the reciprocal nature of classroom ecology

Schools constitute a primary developmental ecology (Kiuru et al., 2020), promoting positive youth development (Eccles and Roeser, 2009) by optimizing student wellbeing, healthy growth, and academic achievement (Darling-Hammond et al., 2020). Accordingly, educators must establish proactive, authoritative leadership within the classroom while simultaneously cultivating supportive relationships, thereby co-constructing an environment in which both teachers and learners can thrive. Crucially, educators are not solely responsible for the architecture of successful classroom environments. While teachers hold the professional responsibility to manage instructional delivery and provide stimulating learning opportunities, students share the responsibility to respect systemic boundaries and utilize these pedagogical resources. Consequently, successful classroom interactions emerge as a joint, transactive responsibility. Through mutually dependent behaviors, educators and students actively shape an interpersonal system while being continuously shaped by it.

**Figure 2:**
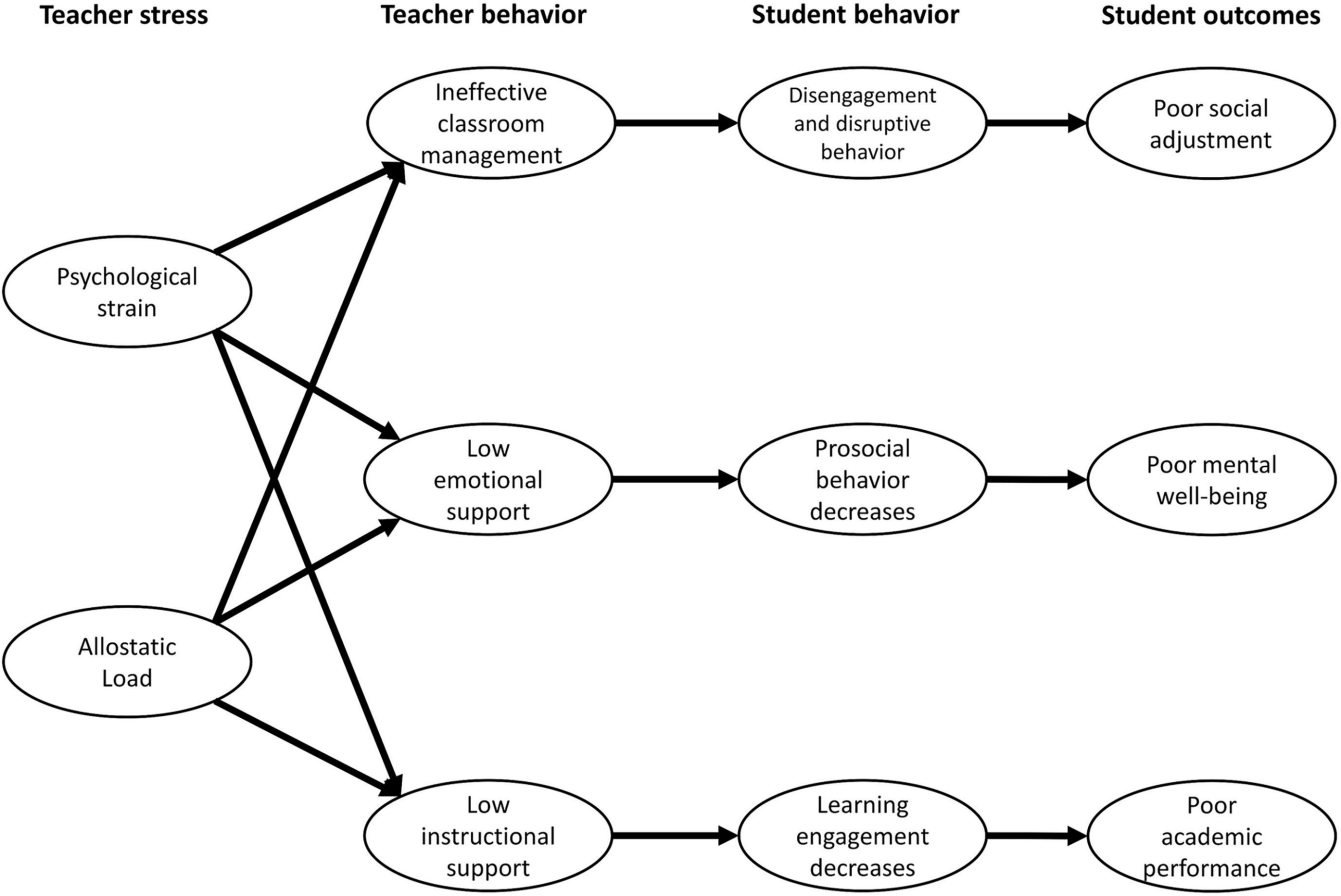
Possible pathways between teacher stress, teacher and student behavior, and student outcomes.

To map this transactional process onto the definitive feedback loops of the ATLAS model (see Figures 1, 2), the classroom ecology must be evaluated through the background between functional and dysfunctional interaction systems.

#### 2.4.2 Functional interaction systems

Functional interaction systems are characterized by effective classroom organization, supportive relationships, and a stimulating learning atmosphere. Effective organization operates as a shared synergy. Educators facilitate this through proactive classroom management, the establishment of predictable routines, and the continuous monitoring of instructional flow, while students contribute through normative behavioral compliance and sustained academic engagement. Within this framework, classroom management encompasses any pedagogical behavior designed to foster an environment that systematically supports both academic and socio-emotional learning (Evertson and Weinstein, 2013). This paradigm emphasizes prevention through environmental planning, integrating key dimensions of structural organization, resource management, and relational discipline (Emmer and Sabornie, 2015). Adaptive classroom management comprises effective monitoring, low-threshold interventions, and rule clarity, which optimize time-on-task and elevate overall learning success. Empirically, such highly adaptive classroom management actively serves as a physiological buffer against teacher biological stress markers (La Marca et al., 2023).

Furthermore, supportive teacher–student relationships (Hamre and Pianta, 2006) are characterized by high levels of both educator agency and communion (Wubbels et al., 2006). A nurturing relational foundation effectively prevents classroom disruptions (Scherzinger and Wettstein, 2022), promotes prosocial student behaviors (Obsuth et al., 2017), and fosters academic motivation (Raufelder et al., 2022), school engagement (Klem and Connell, 2004; Wentzel, 1998), cognitive learning (Vansteenkiste et al., 2005), academic achievement (An et al., 2023), and systemic school adjustment (Sabol and Pianta, 2012). Concurrently, these relationships mitigate student stress experiences and school exhaustion (Hoferichter et al., 2022; Wentzel et al., 2017), validating the Buffering Hypothesis (Cohen and Wills, 1985) and offering critical resources that help individuals master stressors in volatile situations (Conservation of Resources Theory; Hobfoll, 1989). Ultimately, supportive teacher-student relationships exert a powerful protective effect on the educator’s own somatic health (Wubbels et al., 2006), exhibiting robust associations with lower long-term hair cortisol concentrations in teachers (La Marca et al., 2023).

#### 2.4.3 Dysfunctional interaction systems

Conversely, dysfunctional interaction systems are characterized by disrupted classroom organization, fragmented social relationships, and self-reinforcing burnout cascades. Within the ATLAS framework, classroom disruption is conceptualized as an interactional phenomenon rather than an isolated individual deficit, meaning that dysregulation can emanate from both students and teachers (Scherzinger and Wettstein, 2019). Student aggression at school manifests through diverse typologies (Björkqvist et al., 1992) and constitutes one of the most taxing challenges educators face (Chang and Davis, 2009). However, teachers may also engage in aggressive behaviors, such as exposing or unfairly penalizing students (Krumm, 2002). Interpersonal aggression operates as a profound stressor that threatens essential social bonds (Epel et al., 2018). Empirically, a high prevalence of observed student aggression predicts an elevated heart rate and suppressed heart rate variability (HRV) among teachers (Kühne et al., 2024), while a high frequency of such exposure correlates with elevated hair cortisol levels (La Marca et al., 2023).

These dysfunctional dynamics frequently mature into coercive-escalative patterns (mutual fight/fight reactions) or systemic alienation (mutual flight/flight reactions). During acute aggressive episodes, educators experience an exceptionally dense interaction velocity, often exceeding 15 micro-interaction turns per minute (Wettstein et al., 2024). Under these conditions, negative peer norms can stabilize, wherein deviant behavior is rewarded with social status, accelerating the collective stress load. Through these mutually reinforcing negative trajectories, a stable, dysfunctional interaction system emerges.

#### 2.4.4 Pathways between teacher stress and negative student outcomes

We now illustrate how advanced teacher strain and allostatic load compromises pedagogical classroom behavior, thereby triggering maladaptive student behaviors and adverse developmental outcomes, such as compromised social adjustment, impaired wellbeing, and depressed academic performance over time (see Figure 2).

As illustrated by the case of Mia, elevated teacher stress degrades personal coping resources and manifest classroom behavior, thereby undermining student behavioral and developmental trajectories. Consequently, individual psychological strain and high allostatic load do not influence student outcomes via a direct, unmediated pathway; rather, these systemic effects are fully mediated by observable teacher and student behavior. Advanced teacher stress drives ineffective, chaotic classroom management, which subsequently escalates student disengagement and disruptive behaviors, leading to negative social adjustment and compromised learning. Thus, these manifest interactional behaviors act as the critical intermediate step in the transactional pathway linking teacher stress to student development.

Crucially, negative student outcomes feed directly back into the educator’s regulatory network, intensifying teacher stress. To capture these bidirectional, reciprocal relationships over time, empirical research must deploy advanced cross-lagged panel models. Moreover, these transactional associations are powerfully moderated by individual student characteristics, including age, gender, and pre-existing behavioral profiles. Youth exposed to early life adversities or manifest behavioral disorders exhibit heightened vulnerability to compromised teaching practices compared to their less vulnerable peers. Furthermore, meta-analytic data (Tao et al., 2022) reveal that the protective impact of perceived teacher support is significantly more pronounced among upper-secondary students than within elementary cohorts.

The core innovation of the ATLAS model lies in the formalization of these self-reinforcing, bidirectional feedback loops. As formalized in Figure 2, downstream student outcomes do not act as static endpoints. Instead, elevated student disengagement and aggression feed directly back into the left-hand section of the model, presenting severe interpersonal stressors that deplete the teacher’s remaining self-regulatory capacity. This reciprocal escalation solidifies maladaptive peer norms and accelerates the teacher’s biological allostatic load, successfully entrapping the entire classroom environment within a chronic burnout cascade.

## 3 Methodological recommendations for future research

### 3.1 Designs and measurement strategies

Our approach relies on longitudinal ambulatory assessment designs (Wettstein et al., 2021) that incorporate biological measurements, self-reports from both teachers and students, and detailed classroom observations. We assess (1) teachers’ self-reports, (2) teachers’ allostatic load, (3) classroom environment, and (4) student development. By this multimodal approach assessing biological markers, self-reports, and behavioral observations, we examine the intricate, bidirectional relationships among factors contributing to teacher stress and student development. Psychological indicators help us assess the teacher’s previous experiences, habitual mental filters, resources, and vulnerabilities that shape their perception of the environment. These indicators provide a unique insight into the teachers’ adaptive and maladaptive coping attempts. Additionally, they help us understand why some teachers become co-constructors of an increasingly hostile environment. Physiological indicators reveal how psychosocial stress “gets under the skin” and make teachers aware of unfavorable, perhaps unnoticed risks at an early stage. Promising biological markers for assessing the acute stress reaction include salivary cortisol, salivary alpha-amylase, heart rate, and heart rate variability (Wettstein et al., 2021). Allostatic load indicators include cardiovascular (e.g., heart rate, heart rate variability, and blood pressure), neuroendocrine (e.g., hair cortisol), immunological (e.g., markers of inflammation), and metabolic (e.g., body mass index) markers (Juster et al., 2010). Behavioral observations provide a more objective picture of potential stressors than subjective assessments alone. Findings to date indicate that objective indicators of classroom environments explain distinct variance in biological teacher stress, highlighting their value alongside traditional self-reports (La Marca et al., 2023).

### 3.2 Analytical strategies

To capture the complexity of these dynamics, we employ a three-fold analytical approach: cross-lagged panel models to test for reciprocal effects between perceived student aggression and vital exhaustion; latent growth curve models to map individual trajectories of chronic teacher stress; and longitudinal multilevel analyses to investigate the impact of teacher stress on (aggregated) student development. We anticipate that teachers’ allostatic load and psychological strain will increase their perception of potentially stressful situations, impair the quality of their teaching, and ultimately, affect student motivation and performance. However, we expect the associations between psychological variables and biological markers among teachers to be weak. Previous studies indicate that a person’s psychological and biological systems have only weak connections (Stalder et al., 2017). In contrast, we anticipate significant effects of the objective classroom environment on teachers’ biological stress and on students’ social and cognitive development.

### 3.3 Preparing the ground for interventions

Within the ATLAS framework, we collaborate with educators over a two-year timeframe to achieve three primary objectives: (1) to optimize teacher health by identifying proximal risk and protective profiles while modeling the longitudinal relationship between psychological strain and allostatic load; (2) to foster constructive social interactions within the classroom environment; and (3) to facilitate positive student development. To operationalize these objectives, we propose a systematic, three-step intervention protocol:

1. *Screening and individualized diagnostics*: longitudinal multi-method data enable the efficient identification of existing vulnerabilities, psychological resources, and environmental risks. Educators are provided with individualized, multi-systemic diagnostic reports annually, which serve as the empirical foundation for tailored, collaborative discussions regarding targeted interventions specific to their unique professional and physiological circumstances.
2. *Mitigation of cumulative strain*: as illustrated in Mia’s case example, specific educators suffer from elevated psychological strain and allostatic load over extended career intervals. This chronic allostatic overload frequently remains unrecognized, quietly inducing long-term pathophysiological alterations across the cardiovascular, endocrine, metabolic, and immune systems. Furthermore, high psychological strain narrows an educator’s cognitive capacity, distorting their appraisal of student behavior, impairing coping execution, and destabilizing live classroom management. Consequently, strained teachers may unintentionally co-construct an aversive, hyper-reactive classroom climate. Reducing teachers’ cumulative allostatic strain operates as an absolute prerequisite for the successful implementation of secondary pedagogical interventions. The introduction of novel classroom management strategies or the cultivation of complex relational skills demands substantial cognitive and emotional energy; executing these adjustments while in a state of allostatic depletion inevitably exacerbates psychological exhaustion. Thus, addressing existing physiological wear must precede the training of new behavioral repertoires. We recommend a systematic management protocol for these chronic stress states, analogous to the clinical rehabilitation of physiological overload: first, structurally reducing the overall occupational workload while identifying the specific systemic catalysts of the strain; and second, executing targeted behavioral adjustments alongside professional, transdisciplinary healthcare assistance when indicated.
3. *Targeted multi-level training*: once individual diagnostics and systemic strain mitigation have successfully stabilized the teacher’s physiological baseline, structured training protocols can be initiated. These interventions draw upon proven, ecologically validated frameworks, such as the Coping-Competence-Context (3C) Theory (Herman et al., 2020b), which simultaneously addresses individual stress-regulation skills (Coping), proactive, low-threshold classroom management capabilities (Competence), and the cultivation of a supportive, resourceful institutional school environment (Context).

## 4 Discussion

### 4.1 Theoretical contributions and distinctiveness of the ATLAS model

The ATLAS model fundamentally extends existing teacher stress paradigms by opening the psychological “black box” and integrating objective multi-systemic biological markers into an interactive feedback loop. Unlike the traditional Job Demands-Resources model or Lazarus’s Transactional Theory, which rely heavily on subjective self-perceptions, ATLAS accounts for a more objective representation of the classroom ecology as a primary driver of allostatic load.

Our framework advances the field through four distinct propositions:

1. *Psychobiological core:* teacher stress is conceptualized as an integrated psychobiological process rather than an isolated psychological state. This perspective aligns with recent evidence showing that intensive emotional labor directly accelerates biological wear-and-tear and elevates cumulative physiological risk factors (Attwood, 2024).
2. *Interactional systemic ecology*: stress within the ATLAS framework is not viewed as an individual deficit in emotional management. Instead, it is a co-constructed systemic ecology. This allows us to decenter the individual “resilient teacher” and capture how active coping, competence, and instructional quality are continuously shaped by the broader social ecologies and interpersonal dynamics within the classroom (Oldfield and Ainsworth, 2022).
3. *Ecosystem alteration:* chronic allostatic overload systematically shifts the classroom ecology by impairing the teacher’s automated behavioral regulation, leading to structural disruptions in classroom organization.
4. *Objective indicators over self-reports:* the model prioritizes more objective, multi-method observations and physiological indicators, which serve as highly robust predictors of systemic cascades compared to self-reported stress profiles.

By bridging the gap between affective neuroscience and school-based interaction research, ATLAS overcomes the systemic fragmentation that often characterizes the study of occupational strain. Rather than treating psychology and biology as separate strata, the model demonstrates their seamless translation into the relational fabric of the classroom, establishing a truly transdisciplinary language for future intervention designs.

### 4.2 Boundaries and limitations of the ATLAS model

While the ATLAS model provides a comprehensive, transdisciplinary framework for understanding the psychobiological and systemic nature of teacher stress, several structural and ecological boundaries must be considered when applying or generalizing the model.

First, the behavioral thresholds embedded within the model’s interactional core are highly dependent on cultural contexts. What constitutes a “disruption,” “verbal aggression,” or “appropriate teacher agency” varies significantly across global educational systems. Varying classroom interaction norms and baseline institutional structures directly alter how relative stressors are appraised through the “NUTS” criteria (Dickerson and Kemeny, 2004). Consequently, while the underlying biological feedback loops (such as HPA-axis activation) remain universal, the interpersonal friction points and relational display rules governing emotional labor must be calibrated using culturally validated observation protocols.

Second, the model’s feedback loops are heavily moderated by the socioeconomic conditions of the school district and the specific school context (e.g., mainstream vs. special education schools). Schools in low-income areas often face chronic underfunding, higher student-to-teacher ratios, and a higher prevalence of students exposed to early life adversities (Ellis et al., 2022). In these high-adversity contexts, the classroom ecology is exposed to external systemic pressures that can accelerate a teacher’s allostatic overload independently of individual teacher competence. Furthermore, the capacity of the school context to act as a supportive buffer (Herman et al., 2020b) is often constrained by systemic resource deficits, which can exacerbate the speed and intensity of burnout cascades.

Third, macro-level variations across national educational systems impose structural boundaries on the model’s generalizability. Differences in educational accountability policies, standardized testing regimes, and teacher autonomy account for up to 18% of the variance in teacher job stress (Kim et al., 2020). In educational systems with rigid top-down accountability, a teacher’s capacity for adaptive classroom management and proactive regulation is structurally restricted. The ATLAS model primarily captures proximal classroom dynamics, meaning that these distal macro-level institutional pressures must be explicitly treated as powerful contextual boundary conditions.

Key methodological limitations of the framework concern the logistical, financial, and ethical feasibility of multi-systemic biological monitoring in real-world school settings. Collecting continuous ambulatory physiological data (e.g., HRV, salivary markers) and cumulative biomarkers (e.g., hair cortisol) requires substantial financial funding, specialized laboratory access, and extensive participant compliance. These barriers restrict the large-scale implementation of the ATLAS model as a routine diagnostic tool for educational institutions.

To resolve this limitation, the ATLAS model should not be viewed as a mandatory blueprint for continuous bio-monitoring. Instead, it serves as a scientific heuristic designed to validate scalable, non-invasive behavioral proxies. Future empirical research must focus on identifying specific, observable behavioral patterns, such as high interaction density during conflicts or observable relational withdrawal, that strongly correlate with underlying biological allostatic wear-and-tear (La Marca et al., 2023), thereby translating complex psychobiological insights into practical tools for teacher education and school health prevention.

## 5 Conclusion

The “ATLAS – Psychobiological Model of Teacher Stress in Classroom Interactions” introduced in this manuscript integrates various disciplinary approaches within a comprehensive framework. By considering biological, psychological, and social processes across distinct temporal dimensions, the ATLAS Model provides a robust framework for systematically identifying risk and protective factors, tracing the long-term consequences of stress on health and perceptions of the classroom environment, and understanding its direct impact on live classroom interactions and student development. By opening the psychological “black box” and linking biological vulnerabilities, such as acute HPA-axis reactivity and chronic allostatic overload, with manifest interactive behaviors, the ATLAS framework demonstrates that teacher strain, allostatic overload, compromised instructional quality, and negative student outcomes are not linear endpoints but components of a continuous feedback loop.

Although we use a longitudinal ambulatory assessment approach, we advocate for a high degree of openness in research methodologies and interdisciplinary approaches to teacher stress. Therefore, we advocate for combining a diverse array of methodologies rather than pitting them against one another. Embracing this diversity in approaches and disciplines will enhance our understanding and ultimately lead to more effective interventions that can transform the educational landscape.

To advance the ATLAS framework toward its full predictive capacity, future empirical research must address three critical horizons. First, studies should aim for a specification of idiosyncratic biological and psychological tipping points, modeling precisely the personalized thresholds of allostatic load at which an individual teacher’s emotional regulation capacity collapses. Second, future designs should formalize a quantitative taxonomy of interaction density, employing dynamic systems theory to calculate the exact interpersonal frequencies at which aggressive episodes precipitate unyielding burnout cascades. Third, research must systematically operationalize and validate observable behavioral proxies, such as specific verbal patterns or micro-expressions, that can serve as non-invasive, scalable indicators of underlying physiological strain, bypassing the logistical barriers of continuous bio-monitoring in day-to-day school operations.

This holistic, multi-method perspective underscores the need for interventions that extend beyond individual coping strategies and address the broader, dynamic interplay between teacher stress, the classroom environment, and student development. Ultimately, ATLAS moves the field away from placing the burden of resilience solely on the individual educator. Instead, it underscores the systemic imperative of designing multi-level prevention strategies that simultaneously sustain the physiological wellbeing of teachers and restore the relational safety of the classroom ecology, ensuring thriving learning environments for the next generation.
